# Proposing a Practical, Simplified Framework for Implementing Integrated Diabetes Data and Technology Solutions

**DOI:** 10.3389/fcdhc.2022.867284

**Published:** 2022-05-11

**Authors:** Juan C. Espinoza, Steven W. Chin, Payal Shah, Maurice Tut, Jennifer K. Raymond

**Affiliations:** ^1^ Division of General Pediatrics, Department of Pediatrics, Children’s Hospital Los Angeles, Los Angeles, CA, United States; ^2^ Translational Informatics, Information Services Department, Children’s Hospital Los Angeles, Los Angeles, CA, United States; ^3^ Department of Pediatrics, Keck School of Medicine, University of Southern California, Los Angeles, CA, United States; ^4^ Division of Neonatology, Department of Pediatrics, Fetal and Neonatal Institute, Children’s Hospital Los Angeles, Los Angeles, CA, United States; ^5^ Division of Endocrinology, Department of Pediatrics, Children’s Hospital Los Angeles, Los Angeles, CA, United States

**Keywords:** diabetes, technology, implementation, framework, integration, data

## Abstract

Diabetes is a uniquely quantifiable disease, and as technology and data have proliferated over the past two decades, so have the tools to manage diabetes. Patients and providers have at their disposal devices, applications, and data platforms that generate immense amounts of data, provide critical insights into a patient’s disease, and allow for personalization of treatment plans. However, the proliferation of options also comes with new burdens for providers: selecting the right tool, getting buy-in from leadership, defining the business case, implementation, and maintenance of the new technology. The complexity of these steps can be overwhelming and sometimes lead to inaction, depriving providers and patients of the advantages of technology-assisted diabetes care. Conceptually, the adoption of digital health solutions can be thought of as occurring in five interconnected phases: Needs Assessment, Solution Identification, Integration, Implementation, and Evaluation. There are a number of existing frameworks to help guide much of this process, but relatively little attention has been focused on integration. Integration is a critical phase for a number of contractual, compliance, financial, and technical processes. Missing a step or doing them out of order can lead to significant delays and potentially wasted resources. To address this gap, we have developed a practical, simplified framework for integrating diabetes data and technology solutions that can guide clinicians and clinical leaders on the critical steps in adopting and implementing a new technology.

## Introduction

Diabetes is a uniquely quantifiable disease, and as technology and data have proliferated over the past two decades, so have the tools to manage diabetes. Patients and providers have at their disposal devices, applications, and data platforms that generate immense amounts of data, provide critical insights into a patient’s disease, and allow for personalization of treatment plans. However, the proliferation of options also comes with new burdens for providers: selecting the right tool, getting buy-in from leadership, defining the business case, integration with existing systems, implementation, and maintenance of the new technology. The complexity of these steps can be overwhelming and sometimes lead to inaction, depriving patients and providers of the advantages of technology-assisted diabetes care.

Digital health has enormous potential to positively transform the landscape of diabetes management. Ever expanding, the field encompasses a broad set of concepts and technologies, including telehealth, mobile applications, wearables, artificial intelligence, and precision medicine (1–3). The ongoing push to reach this potential has led to a staggering proliferation of digital health solutions. In 2021, more than 350,000 digital health applications are currently available to consumers, with 90,000 of these introduced just since 2020 (4). Funding for digital health companies reached $57.2 billion in 2021, an increase of 79% from 2020 (5). This rapid growth has been paired with a relative lack of standardized frameworks aiding with solution discovery, clinical integration and evaluation. This leaves stakeholders with challenges in planning and execution of an effective implementation of a digital health solution. High profile examples of problematic implementations have demonstrated that the implementation of an intervention is as important as the intervention itself in achieving the desired end goal (6–9).

**Figure 1:**
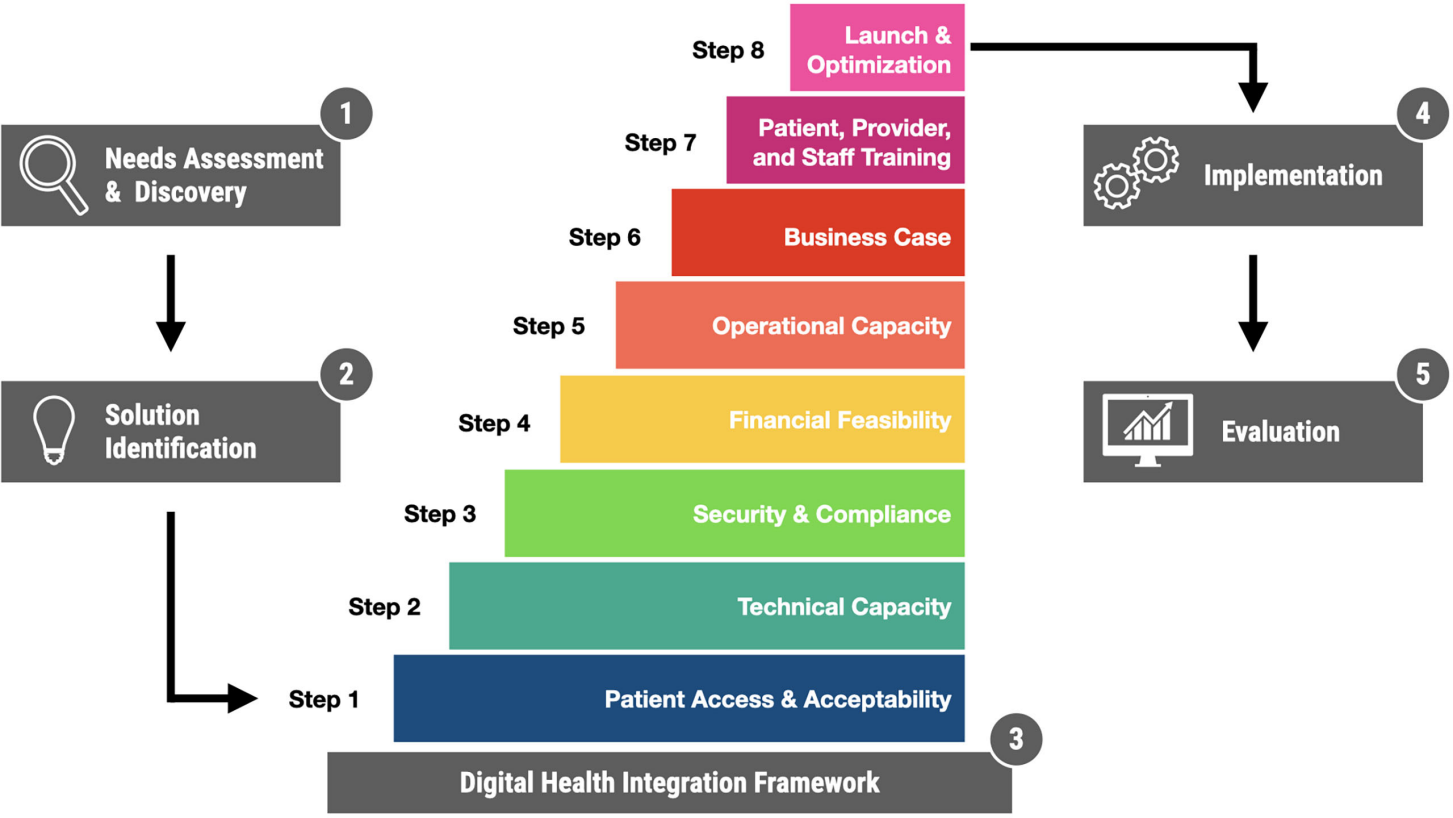
Digital Health Solution Adoption with a focus on Integration. The process is presented here in five sequential phases, with detailed integration framework presented in eight steps.

Conceptually, the adoption of digital health solutions can be thought of as occurring in five interconnected phases (**Figure 1**):

1. Needs Assessment and Discovery: What is the problem? What is the opportunity? Who are the relevant stakeholders? How does it impact them? What possible solutions exist?
2. Solution Identification: an informal or formal process (such as a request for proposals), through which designated stakeholders evaluate and ultimately select a specific solution.
3. Integration: Bringing the selected technology into alignment with existing technical systems and operational practices.
4. Implementation: Deploying the technology into clinical practice.
5. Evaluation: Ongoing monitoring of outcomes, with a focus on safety, effectiveness, quality improvement, value, and research objectives.

There are a number of existing frameworks to help guide much of this process, but relatively little attention has been focused on integration (phase 3). In this article, we provide a brief overview of common approaches to various phases in this process, and describe the integration framework we have developed at our institution.

## Needs Assessment and Discovery

Within the context of healthcare delivery, a needs assessment is the process by which individuals or teams gather and analyze information about the needs of a specific patient population in order to understand the gaps in their care, the causes of those gaps, and how those gaps might be closed (10). Several research and operational needs assessment frameworks have been developed and published by organizations like the CDC (11), WHO (12), NHS (13), Johns Hopkins University (14, 15), the University of Kansas (16, 17), the American Medical Association (18), and the Agency for Healthcare Research and Quality (19).

Health equity is a critical component of any Diabetes-related needs assessment. Diabetes prevalence, morbidity and mortality disproportionately impacts low-income communities and communities of color (20). Social determinants of health (SDoH) are principal drivers of these disparities, and the ability to access technology and the internet is increasingly being recognized as a contributing factor to healthcare disparities; a concept referred to as the digital divide (21). Thus, every healthcare organization considering digital health solutions should be keenly aware of the impact they have on vulnerable populations and their ability to meaningfully access and receive technology-enabled care.

## Solution Identification

Identifying solutions is often an iterative process, and at healthcare institutions, it is sometimes conducted through a formal Request for Proposals (RFP), typically managed by Information Technology (IT) Services. The growing number of emerging solutions leaves organizations the increasingly difficult task of discerning effective, high quality and high value solutions from a crowded landscape. There is a growing body of standards-based frameworks and resources to assist stakeholders in identifying optimal digital health interventions, including:

- National Health Service (NHS) Apps Library (22) - Evaluates digital health solutions across the board in areas of clinical effectiveness, safety, privacy, usability, accessibility and interoperability.
- Wellocracy (23) - Sponsored by Partners Connected Health. Focuses on consumer wellness and self help. Qualitative reviews, no objective evaluation.
- Personalized Healthcare Connected Alliance (24) - 240 provider, payer, pharma, medical device stakeholder groups. Focus on mobile platform interoperability, connected devices, FHIR data standards.
- RankedHealth (25) - MIT Hacking Medicine Institute. Crowdsourced medical technology professionals perform peer reviews of wide range of apps related to heart disease, diabetes, obesity, pregnancy, reproductive health and more.
- Node.Health (26) - Network for Digital Evidence in Health comprised of 20 health systems, accelerators, startups. Creating an evidence-based medicine movement for digital health solutions. Scores apps in areas of clinical efficacy and usability.

## Implementation

A number of implementation theories and frameworks already exist that can be used to guide diabetes technology implementations, such as Normalization Process Theory (NPT) (27, 28), Consolidated Framework for Implementation Research (CFIR) (29–31), Reach Effectiveness Adoption Implementation Maintenance (RE-AIM) Framework (32), and Proctor’s Framework for Implementation Research (33). Nelson et al. published a narrative review in 2020 that provides an overview of various implementation frameworks used in technology-delivered diabetes self-care interventions (34). Many of these implementation frameworks are comprehensive and include, at least in part, aspects of needs assessment, integration, implementation, and evaluation. However, they are principally focused on the clinical implementation, and less so on the technical work required to onboard and integrate a new technology into a clinical setting.

## Evaluation

Evaluation of digital health solutions presents a unique challenge compared to other interventions. The speed of software development and rapid iteration cycles are inherently mismatched with the length and time-cycle of randomized controlled trials or most means of traditional evidence generation (35, 36). This incongruence has produced a digital health evidence gap, resulting in low levels of rigorously evaluated applications and a lack of objective information for stakeholders to accurately assess an interventions usability, functionality, safety, effectiveness, accessibility and value. This gap is being addressed with an increasing body of guidance and proposed frameworks addressing the evaluation of digital health solutions, from governmental agencies, industry-based consortiums and academic institutions (35):

- Monitoring and Evaluating Digital Health Interventions (WHO) (37): a comprehensive framework for monitoring, evaluation and validation at all points in product life cycle.
- Evidence Standards Framework for Digital health and Care Technologies (UK National Institute for Health and Care Excellence) (38): Guidance on evidence generation for effectiveness and economic standards. Divides guidance into functional categories based on three tiers of digital function.
- Continua Design Guidelines (Personalized Connected Healthcare Alliance) (39): Framework of standards and criteria required to ensure interoperability of apps focused on personal health and wellness.
- Non-adoption, Abandonment, Scale-up, Spread and Sustainability (NASSS) Framework (40): Evidence based framework for prediction and evaluation of digital health solution success based on performance in seven domains.
- Digital Health Scorecard (41): Quantitative scoring of mobile app performance in 5 key domains: technical, clinical, usability, cost, and satisfaction of end user requirements.

## The Missing Piece - Integration

Integration is the process of aligning a new technology with existing technical systems and operational processes. This process has multiple stakeholders from across an organization, ranging from legal and compliance, to IT services, to clinic staff. At large institutions, there may be Project Management Offices (PMO) who are responsible for shepherding new integrations through multiple governance and approval committees, but even then, the process can be complex. During this phase, the contractual, compliance, financial, and technical realities of a project come into sharp focus. Missing a step or doing them out of order can lead to significant delays and potentially wasted resources. To address these issues, we have developed a practical, simplified framework for integrating diabetes data and technology solutions that can guide clinicians and clinical leaders on the critical steps in adopting and implementing a new technology, based on our experience (36, 42–45). This model does not replace but rather complements the previously mentioned implementation frameworks, which pick up where our model leaves off.

Our framework (**Figure 1**) consists of eight sequential steps, presented in **Table 1** with a brief description and a series of questions project teams should ask themselves. To illustrate the framework in action, we present a brief case study based on our local experience.

**Table 1:** A Practical Digital Health IntegrationFframework in Eight Steps.

Step Description and Goals	Key Questions
1.) Patient Access And Acceptability: Before going through the complex integration process and potentially wasting time and resources, first and foremost the obligation is to ensure that the solution in question is appropriate for your patient population. This may be your last opportunity to verify the problem- solution fit from the perspective of your patients.	*Does the select solution work within the constraints of your patient population?* *Will it exacerbate existing barriers to care? Will it create new ones?* *How well does the specific solution align with the findings of your needs assessment?* *Do you need to go back and show the solution you selected to your original stakeholders if they were not part of the solution selection process?*
2.) Technical Capacity: Ensure that your organization has the adequate technical capacity to leverage the solution, including EHR integration, data exchange, account linkage, hardware, and connectivity.	*Does the solution require integration into your EHR or other systems?* *Do you need new or specific hardware, like tablets or unique connectors?* *Will you need to add hardware like second monitors or bar code scanners to allow your staff to take advantage of all the features of the technology?* *Do you have adequate internet access for patients and providers?* *Does the solution use existing interoperability and data standards?* *Do you have the right technical talent in-house, or will this require a third-party vendor to support integration?*
3.) Security and Compliance: There are a number of laws and regulations that govern the exchange of patient data. Your institution’s legal and compliance department will need to be involved early on to review all contracts and service agreements. Information security review is critical to ensure patient privacy and reduce risk and liability.	*What type of legal agreements need to be in place to cover your specific use case? For example, in the US, exchanging health data with a third party requires a business associate’s agreement (BAA) to be in compliance with the Health Insurance Portability and Accountability Act (HIPAA) (44).* *Have you performed an information security assessment?* *What information will you have to share in order to link patient accounts across systems?* *Will patients need to provide consent, or is this covered under other permissions and consents?*
4.) Financial Feasibility: The true cost of a solution may not be known until you start the integration process, as there may be explicit charges from the vendor related to integration, or internal costs of buying hardware or IT Services time to build integrations. This stage is intended to evaluate the total project costs (purchase, installation, maintenance) and whether or not your institution or organization is prepared to shoulder those costs.	*Are there integration or deployment fees?* *Are there training fees?* *Are their maintenance fees or recurring monthly or annual fees?* *Is customer support included? What about for patients?* *What is the hourly rate for technical support?* *What are the payment terms? Does the vendor expect payment within a specific timeframe? Can your organization meet that timeframe?*
5.) Operational Capacity: Almost all new technologies require some kind of workflow, whether its patient enrollment, education, account connection, data extraction etc. You will need to consider how these new workflows will be incorporated into the daily activities of existing team members, or onboarding new staff to add capacity.	*Do you have a complete lists of all key processes related to the new technology?* *Can you map all those processes to existing team members? Do you need additional staff?* *How do those new processes compliment, shift, or interfere with existing responsibilities?* *How will you assign these responsibilities?* *Onboarding a new technology is labor intensive; much more so than the actual technology workflow. Do you have additional support during the launch* *phase? If not, what will you de-prioritize?* *Is your staff aware of this coming change? How has it been communicated?*
6.) Business Case: New technologies may facilitate new revenue opportunities, such as remote patient monitoring, chronic care management, data review, etc. They may also help practices achieve certain value-based or quality metrics that also result in financial incentives. Finally, new technologies can also result in operational efficiencies that reduce overhead costs. All of these need to be considered and business cases must be made for the implementation of a new technology.	*Will you be introducing new billing codes? Have they been added to your billing software? Have you trained your clinical and administrative staff on how to use them?* *Have you reviewed the requirements and documentation needed to successfully bill these new codes?* *Are there copays for patients? How will you collect them? How will you let them know that there may be new financial burdens associated with using this technology?* *How does this new technology affect your other services and revenue streams?* *How will you track savings or value to justify the cost of the new technology?*
7.) Patient, Provider, and Staff Training: All users should be made aware of the new technology, the reason for its implementation, what benefits they should expect, and how its implementation will impact their current care or workflows. Training and training resources are critical to any successful implementation.	*Does the vendor provide training resources? In what languages? How are they accessed? If online, can you reach their website from your network?* *Are there also quick reference tools to use during deployment?* *Will you need to develop any additional training resources specific to your environment?* *How will you distribute training materials to patients? Are the culturally and linguistically appropriate? Are they at an appropriate literacy level?*
8.) Launch and Optimization: The actual deployment of the technology should be carefully planned, monitored, and evaluated. Existing implementation and evaluation frameworks can all be excellent tools to guide this step in detail. Broadly speaking, the acronym SEQVR (pronounced “secure”) can help organize and prioritize evaluation: Safety, Effectiveness, Quality Improvement, Value, Research.	*Which Implementation Framework will you use?* *Have you identified a leader and team who will oversee implementation?* *Have you established a timeline?* *How will you know you’re done with implementation?* *What are your key outcomes and metrics for evaluation?* *Have you selected an Evaluation Framework?* *How will you share your evaluation results with key stakeholders?* *How will evaluation inform your own practice?*

## Case Study: Continuous Glucose Monitoring Integration

Hospital System EPTR has identified a gap in the management of their diabetes population using Medtrix continuous glucose monitoring (CGM) devices. An internal root cause analysis pointed to a lack of available structured CGM data/metrics in the EHR has increased the time needed to document clinical notes, creating additional administrative burden on clinicians. To address this, it has been recommended that EPTR create a CGM data integration with Medtrix. From a patient perspective, Medtrix is the most commonly used CGM in our patient population. Many patients do not have access to the internet at home, so they upload their data during clinic visits using hospital computers or hospital wifi. This process is time consuming for patients and staff, and also needs to be addressed.

With this recommendation, EPTR clinical and IT leadership began to assess the feasibility/capability in planning and implementing a CGM data integration. The planning consisted in evaluating the technical, security and legal, financial, and operational considerations in play.

To assess the technical capacities of EPTR, IT leadership requested and reviewed integration strategy documentation from Medtrix. During technical calls with Medtrix, it was determined that EPTR’s current capability to send and receive EHR HL7 messages externally through a third-party integration vendor, made EPTR a great candidate for this type of integration. For providers, they would continue to use the existing computerized physician order entry (CPOE) function to request CGM data within the EHR.

With recent security breaches within the healthcare industry, EPTR’s information security and legal teams have implemented a variety of processes to mitigate liability and risk. EPTR’s IT security team requested comprehensive technical details of Medtrix’s IT security posture and data management. In addition to executing the vendor’s Data Subscription Agreement, detailing the services and cost of the integration, a separate security addendum provided by EPTR was added. The addendum ensures Medtrix has industry standard security practices and maintains certain service levels. With Medtrix’s status as a hybrid covered entity under HIPAA, no Business Associate Agreement needed to be executed. From a legal perspective, the type of data provided through the integration service falls under the Treatment, Payment, and operations clause, but to decrease liability, data is requested *via* CPOE orders and not pushed automatically to the EHR.

EPTR’s financial assessment consisted of three considerations: Medtrix’s annual data subscription fee of $8,000, internal project team labor cost, and cost of maintaining the integration. Much of the project team’s cost will come from IT design/development work and testing of the HL7 messages and CPOE order sets. Maintenance cost was determined to remain relatively low. Any additional data being requested through the CGM integration can be worked on by EPTR’s internal clinical systems and integration team.

Even though the main objective of a CGM data integration is meant to reduce or eliminate repetitive administrative workflows, it does introduce new ones. Previously, clinical staff was tasked with logging into Medtrix’s data platform to download, print out an AGP report, hand it to the physician, and scan the document into the EHR. With the new CGM data integration, clinical staff were now tasked with submitting CPOE orders to ensure patient consent and account linkage. Once consent is provided by the patient *via* email, clinical staff can submit a secondary CPOE order to request CGM data for a particular timeframe. Fortunately, no additional staff was needed to accommodate this new workflow but did require adjustments to clinic workflow.

After a comprehensive review of the feasibility of this effort, clinical and IT leadership created a business case to present to EPTR’s capital committee. Not only was the business case essential to requesting capital funds to implement the project, it also provided the institution the rationale of how this new technology was beneficial to their patients and clinicians. The benefits consisted of the following: remote patient monitoring within the EHR, improve clinical documentation with structured glucose metrics (see trend analysis in a flowsheet/results review), eliminate need to print out and scan reports, use structured data for population health management, help achieve value-based metrics, and may provide additional reimbursement.

Once the budget was approved and contracts were executed, a project manager was assigned to implement the data integration. A project charter was developed to provide a detailed project plan and to receive approvals from the various stakeholders involved. After assigning project resources internally from clinical and IT dept, a project kick-off call was held with Medtrix’s project team. It was at this stage where a communication campaign was created, to make providers and staff aware of the implementation of the CGM integration. This allowed the Endocrinology dept and its staff to understand the new technology, but also become aware of the change in their workflow.

The actual implementation of the CGM data integration was carefully planned out and documented within the project charter. For the design phase, the clinical project team worked to define the glucose metrics and the data timeframe preferred. Once defined, IT resources began to work on defining the HL7 messaging to send and receive data for the integration. In addition, a CPOE order set was also designed to allow clinicians to order the sending of patient consents *via* email to patients and orders for 14, 30, 45, 90 days of glucose data. Clinical staff were also engaged to map out their existing workflow and work to define a new one with the new integration in place. Within the execution phase, the various components were tested to ensure data flow between EPRT EHR and Medtrix. Clinical staff were asked to run through the new workflow within a test environment.

Before go-live, 3 weeks were dedicated to developing training guides and providing virtual or in person training on the new technology and workflow. Go live support was also provided on the day of and 2 weeks after. To ensure project success and identify potential improvements, a report was created to track the usage of the CPOE CGM order sets, and the amount of data transferred through the integration. A dashboard was also created to help with population management using the structure data.

## Discussion

Technical and operational integration are critical for the successful implementation of any digital health project. For experienced teams and institutions with robust project management practices, these may be second nature, but even at the most innovative institutions there can be oversights and failures. In the same way that checklists have improved many aspects of safety and quality across healthcare (indeed, across most industries), team may wish to create their own local checklists of processes, individuals, and governing bodies that correspond to the 8 steps in the integration framework, and systematically implement it for all digital health projects.

Some of the components of our proposed Integration Framework may sound familiar or even redundant to teams familiar with the needs assessment, implementation, and evaluation frameworks briefly reviewed here. This was done deliberately; many of these steps, if not addressed at this stage, can lead to critical system failures during implementation and evaluation, resulting in failed implementation, wasted funds and effort, possible patient harm, and liability exposure. For teams interested in implementing our approach, we do not recommend duplicating work or steps, but rather consider the process from beginning to end across all 5 phases, selecting their preferred frameworks, and then mapping the steps of the integration framework to their cognates in other frameworks. This will allow teams to address each step comprehensively in the appropriate phase (e.g., Step 1, ensuring patient access and acceptability, may already be part of your needs assessment or solution identification).

In our experience, many diabetes data and technology solutions do not have explicit patient processes, workflows, or technical capacity requirements, which leads to implementations that are frustrating for the patient, family, provider, and care team. Our hope is that following the outlined steps will facilitate the adoption of technologies that increase patient engagement and empowerment in regard to accessing and reviewing diabetes data. Many patients with diabetes don’t currently have the ability or tools needed to access their data. Not only will increased access improve current care, patients will learn life-long skills related to diabetes care and improve self-efficacy. Finally, successful digital health implementations can enable improved patient care in synchronous and asynchronous models.

There are limitations to the integration framework. It represents a single institution’s experience and has not undergone more robust vetting. It is intentionally generic to accommodate a variety of data and technology solutions, but this means that more detailed information for specific modalities, such as telehealth, continuous glucose monitoring, and school-based care, is not covered. It also assumes that an organization has people and processes in place to address many of the steps discussed (for example, information security assessment), and as such may not be as helpful in resource-limited settings. Finally, this framework assumes local or institutional management of resources; the framework may need to be adapted to meet the needs of large health systems or countries with national public health systems.

Despite these limitations, we believe that following this practical framework can help reduce friction, prevent delays, and avoid potentially disastrous oversights during the technology selection and implementation process. We hope that others find this tool useful in their own implementations of diabetes data and technology.

## Author Contributions

JE conceived the manuscript, wrote the first draft, and developed the figures. SC, PS, MT, and JR contributed to the conception and refinement of the manuscript, as well as to writing sections of the manuscript. All authors contributed to manuscript revision, read, and approved the submitted version.

## Funding

This work was supported by the Food and Drug Administration under award number P50FD006425 for The West Coast Consortium for Technology & Innovation in Pediatrics, and by grants UL1TR001855 and UL1TR000130 from the National Center for Advancing Translational Science (NCATS) of the U.S. National Institutes of Health. The funding sources had no involvement in the development of this manuscript or in the decision to submit the paper for publication. The content is solely the responsibility of the authors and does not necessarily represent the official views of the FDA or the NIH.

## Conflict of Interest

JE is a paid consultant for AI Health.

The remaining authors declare that the research was conducted in the absence of any commercial or financial relationships that could be construed as a potential conflict of interest.

## Publisher’s Note

All claims expressed in this article are solely those of the authors and do not necessarily represent those of their affiliated organizations, or those of the publisher, the editors and the reviewers. Any product that may be evaluated in this article, or claim that may be made by its manufacturer, is not guaranteed or endorsed by the publisher.
